# Trimethylsilane Plasma-Nanocoated Silver Nanowires for Improved Stability

**DOI:** 10.3390/ma17153635

**Published:** 2024-07-23

**Authors:** Yixuan Liao, Ganggang Zhao, Yun Ling, Zheng Yan, Qingsong Yu

**Affiliations:** 1 Department of Mechanical and Aerospace Engineering, University of Missouri, Lafferre Hall, Columbia, MO 65211, USA; yltpf@mail.missouri.edu (Y.L.); g.zhao@missouri.edu (G.Z.); ylh3f@mail.missouri.edu (Y.L.); 2 Department of Chemical and Biomedical Engineering, University of Missouri, Columbia, MO 65211, USA; yanzheng@missouri.edu

**Keywords:** plasma nanocoatings, silver nanowires, electrical stability, passivation coatings, humidity effects

## Abstract

The objective of this study was to evaluate the effectiveness of trimethylsilane (TMS) plasma nanocoatings in protecting silver nanowires (AgNWs) from degradation and thus to improve their stability. TMS plasma nanocoatings at various thicknesses were deposited onto AgNWs that were prepared on three different substrates, including glass, porous styrene-ethylene-butadiene-styrene (SEBS), and poly-L-lactic acid (PLLA). The experimental results showed that the application of TMS plasma nanocoatings to AgNWs induced little increase, up to ~25%, in their electrical resistance but effectively protected them from degradation. Over a two-month storage period in summer (20–22 °C, 55–70% RH), the resistance of the coated AgNWs on SEBS increased by only ~90%, compared to a substantial increase of ~700% for the uncoated AgNWs. On glass, the resistance of the coated AgNWs increased by ~30%, versus ~190% for the uncoated ones. When stored in a 37 °C phosphate-buffered saline (PBS) solution for 2 months, the resistance of the coated AgNWs on glass increased by ~130%, while the uncoated AgNWs saw a ~970% rise. Increasing the TMS plasma nanocoating thickness further improved the conductivity stability of the AgNWs. The nanocoatings also transformed the AgNWs’ surfaces from hydrophilic to hydrophobic without significantly affecting their optical transparency. These findings demonstrate the potential of TMS plasma nanocoatings in protecting AgNWs from environmental and aqueous degradation, preserving their electrical conductivity and suitability for use in transparent electrodes and wearable electronics.

## 1. Introduction

Recently, nanomaterials have gained significant attention due to their unique properties in various fields, including optics, mechanics, thermal conductivity, and electrical conductivity [1,2,3,4,5,6,7,8]. Among these nanomaterials, silver nanowires (AgNWs) have garnered particular interest due to silver’s excellent thermal conductivity (429 W/M·K) and high electrical conductivity (6.3 × 10^7^ S/m) [9,10]. Previous studies have demonstrated the potential of AgNW films as an alternative to conventional indium tin oxide (ITO) films for transparent conducting electrodes (TCEs) due to their high optical transparency [11,12,13]. The limitations of ITO films, such as brittleness, high operating temperature requirements, and expensive production costs, have driven the exploration of alternative metal-containing nanomaterials [14]. Metals like silver, copper, gold, titanium, platinum, zinc, magnesium, and iron have been investigated as potential alternatives. In this study, the focus was on AgNWs, which find applications in diverse fields such as solar cells, touch panels, light-emitting diodes (LEDs), and flexible electronics. The unique properties of AgNWs make them promising candidates for various technological advancements, offering advantages over traditional materials in terms of flexibility, cost-effectiveness, and optical transparency [15].

AgNWs have been extensively studied as transparent electrodes on glass substrates [16,17,18]. However, recent research has also explored their application on porous SEBs to simulate on-skin electronics for applications like electrocardiograms (ECGs) and electromyograms (EMGs) [19]. Despite their application potential, AgNWs are prone to oxidation and corrosion due to their nanoscale network structure, leading to decreased conductivity and device performance over time. This has prompted the investigation of various methods to maintain the long-term stability of AgNW-based electronic devices [17,20,21,22]. Different approaches have been explored to prevent oxidation and corrosion in AgNWs. One method involves the production of a hybrid transparent electrode by combining silver nanowires with reduced graphene oxide (AgNW-rGO) [17]. Another approach involves incubating the AgNWs in hydrogen chloride (HCl) vapor to eliminate the oxidized surface [23]. Incorporating polyethoxysiloxane (PES) into the AgNW layer has been reported to enhance oxidation resistance and thermal shock resistance [24]. Fluorocarbon plasma treatment has also been investigated to improve stability against oxidation in high-humidity environments [25]. However, the search for the optimal method to prevent oxidation/corrosion and ensure the long-term stability of AgNW-based electronic devices is ongoing. Further research is needed to identify the most effective approach to address this challenge.

Low-temperature plasma technology offers a versatile approach for surface modification and ultra-thin film deposition, with applications ranging from medical to industrial fields. It allows for chemical reactions between various plasma species and the treated substrate surfaces without significantly affecting its bulk properties. Notably, plasma thin film deposition, known as plasma polymerization, can be carried out at room temperature, ensuring minimal impact on the physical properties of the materials [26,27]. In a previous study, C_4_F_8_ and C_4_F_6_ gases were used in plasma treatment to form thin plasma polymer coatings on AgNWs’ surfaces. However, these thin plasma polymer coatings contained fluoride, which may pose toxicity concerns when AgNW-based devices are applied to on-skin applications [25]. Therefore, it is crucial to explore alternative plasma coating materials that provide effective protection for AgNWs from oxidation/corrosion while ensuring biocompatibility and safety for human skin contact.

In this study, TMS plasma nanocoatings were applied to AgNWs to improve their stability against degradation caused by oxidation and corrosion, particularly under conditions of exposure to humid air or in aqueous solutions such as a PBS solution. The hypothesis of this study is that TMS plasma nanocoatings, a novel technology in comparison to other costly and complex methods, can effectively passivate AgNWs and thus prevent them from oxidation and corrosion.

## 2. Materials and Methods

### 2.1. AgNW Preparation

Porous SEBS substrate preparation: SEBS powders (H1062; Asahi Kasei, Tokyo, Japan) with a weight of 6 g were dissolved into 100 mL of chloroform (purity ≥ 99%) for 1 to 2 h on a magnetic stir plate to make a stock solution. The stock solution was mixed with isopropyl alcohol (IPA, purity ≥ 99.8%)) in a 5:2 volume ratio. The well-mixed solution was then coated onto aluminum foil and dried under ambient conditions overnight to complete the multiscale porous SEBS substrate preparation.

Glass preparation for transparent electronic devices: Cover glasses were selected and applied as transparent electrodes. The cover glasses were cut into approximately 10 mm by 10 mm squares, ultrasonically washed with IPA for 1 min, and then air-dried.

PLLA substrate preparation: PLLA was purchased from Goodfellow Cambridge Ltd., Huntingdon, UK and applied as the substrate of biodegradable electronic devices. The PLLA was cut into approximately 10 mm by 10 mm squares and sterilized with ethanol.

AgNW spray: The AgNWs (Ag NW-D40; ACS Material, Pasadena, CA, USA) used in this study had a diameter of 40 nm and length of 20–60 µm, with a silver purity of >99.5%. A total of 1 mL of an AgNW solution was diluted with 9 mL of IPA and ultrasonicated for 10 s to mix the solution well. Then, the AgNW solution was sprayed onto the pre-stretched multiscale porous SEBS substrates (pre-strain 150%), 10 cm × 10 cm glass substrates, and 10 cm × 10 cm PLLA substrates, using a commercial airbrush (G222; Master Airbrush, CA, USA) through a stencil mask under ambient conditions, with an airbrush pressure of ~20 psi and ~10 cm distance between the nozzle and the substrates. The AgNW-sprayed substrates were rinsed with water and ethanol and then dried at 60 °C in an oven overnight to complete the sample preparation.

### 2.2. Plasma Nanocoating Deposition

The plasma reactor used in this study was an 80 L bell jar plasma reactor. In the plasma reactor, a stainless-steel sample holder plate was positioned between two titanium plates with 10 cm of spacing. The two outer titanium panels served as the electrically grounded anodes, and the stainless-steel sample holder plate served as the powered electrode of the plasma reactor. The AgNW samples were attached to the stainless-steel sample holder. The electrodes were connected to a 13.56 MHz RF power source (RFX-600, Advanced Energy Industries, Inc., Fort Collins, CO, USA) to generate plasmas.

Before starting the plasma processes, the plasma reactor was first vacuum-sealed and evacuated to the base pressure of 1 mTorr. Then, argon gas was first introduced to the plasma reactor with a 1 sccm (standard cubic centimeters per minute) gas flow rate using a mass flow controller (MKS Instruments, Andover, MA, USA) and an MKS 247C readout. The pressure in the plasma reactor was set to be stable at 50 mTorr using the MKS pressure controller. Then, the argon gas plasma was generated with an RF power of 20 W to clean sample surfaces for 1 min. The argon plasma was used as a pre-treatment to remove organic contaminations on the surface of the samples. After the plasma pre-treatment, the reactor was again pumped to the base pressure (1 mTorr) before trimethylsilane (TMS) (Gelest Inc., Morrisville, PA, USA) was introduced to the plasma reactor with a 1 sccm flow rate. The pressure in the reactor was set and stabilized at 50 mTorr. TMS plasma was generated with an RF power of 30 W to deposit TMS plasma nanocoatings. In this study, a 15 s, 1 min, and 2 min TMS plasma-coating time was used to deposit TMS plasma nanocoatings on AgNWs with different coating thicknesses, which were measured as described in Section 2.3.

### 2.3. Plasma Coating Thickness Assessment

The thickness of the TMS plasma coating on the AgNWs was determined based on the measurements of the TMS plasma coating on the silicon wafer coated with the AgNWs at the same time. The plasma nanocoating thickness was measured by using a microscope-mounted thin-film measurement device (Filmetrics F40-UV, KLA Corporation, Milpitas, CA, USA). The thickness range that the device is capable of measuring is 4 nm–40 µm. A small sample area was non-destructively analyzed by reflecting light off the nanocoating. The reflectance spectrum was analyzed over a wavelength range of 200–2000 nm. The FILMeasure 9 software performed curve fitting of the reflectance spectrum to determine the nanocoating thickness.

### 2.4. Sheet Resistance and Optical Transmittance Measurement

To determine if the plasma coating had a negative effect on the resistance of the AgNWs, the electrical resistance of the AgNWs was measured before and after the plasma coating. The optical transmittance variation of the AgNWs on glass was measured after plasma coating. A transmittance analysis was conducted by a Cary 60 UV-Visible spectrophotometer (Cary 60 UV-Vis spectrophotometer, Agilent, Santa Clara, CA, USA).

Electrical resistance was measured for the uncoated and plasma-nanocoated AgNWs. The variation of the resistance change was recorded for 60 days in ambient air in both summer and winter, and in a 37 °C PBS solution.

### 2.5. Surface Wettability Evaluation

Water surface contact angles were measured to evaluate the effect of the plasma nanocoating on the surface wettability of the AgNWs. The measurement was performed by depositing 0.5 µL deionized water droplets onto the surfaces of the uncoated or plasma-coated AgNWs using a computer-aided VCA 2500 XE video contact angle system (AST Products, Inc., Billerica, MA, USA).

### 2.6. SEM and EDS Analysis

The surface morphology of the AgNWs on SEBS substrates with and without TMS plasma nanocoating was analyzed by a field-emission scanning electron microscope (SEM) (Phillips XL30, FEI, Hillsboro, OR, USA). An energy-dispersive X-ray spectroscopy (EDS) was used to detect the surface composition.

### 2.7. Statistical Analysis

Statistical analyses were performed with OriginLab (OriginLab Software Version 2021). The coating thickness and the percentage of resistance increase were presented as mean ± standard deviation (SD).

## 3. Results

### 3.1. Resistance and Transmittance of AgNWs Right after TMS Plasma Nanocoating Deposition

**Figure 1:**
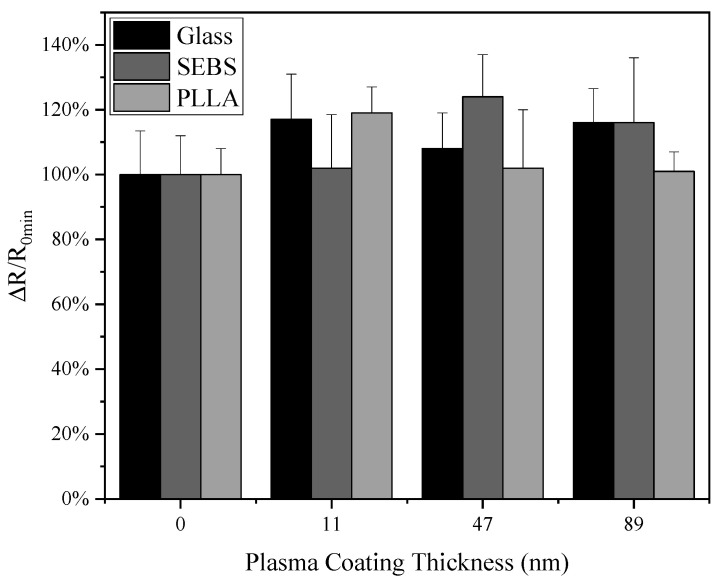
Electrical resistance change percentage of AgNWs on three types of substrates before and right after TMS plasma nanocoating with different coating thicknesses.

The resistance of the AgNWs was measured before and right after the TMS plasma nanocoating deposition. It can be seen from Figure 1 that the electrical resistance of the AgNWs had slightly increased values. With TMS plasma nanocoatings, the highest resistance increase was about 15.5 ± 1.6% (from 3.2 Ω to 3.7 Ω) for the AgNWs on glass substrates, 24.9 ± 2.2% (from 2.4 Ω to 3.0 Ω) for the AgNWs on SEBS substrates, and 16.7 ± 1.4% (from 2.2 Ω to 2.6 Ω) for the AgNWs on PLLA substrates, as compared with the AgNWs on the same substrates without TMS plasma nanocoatings.

**Figure 2:**
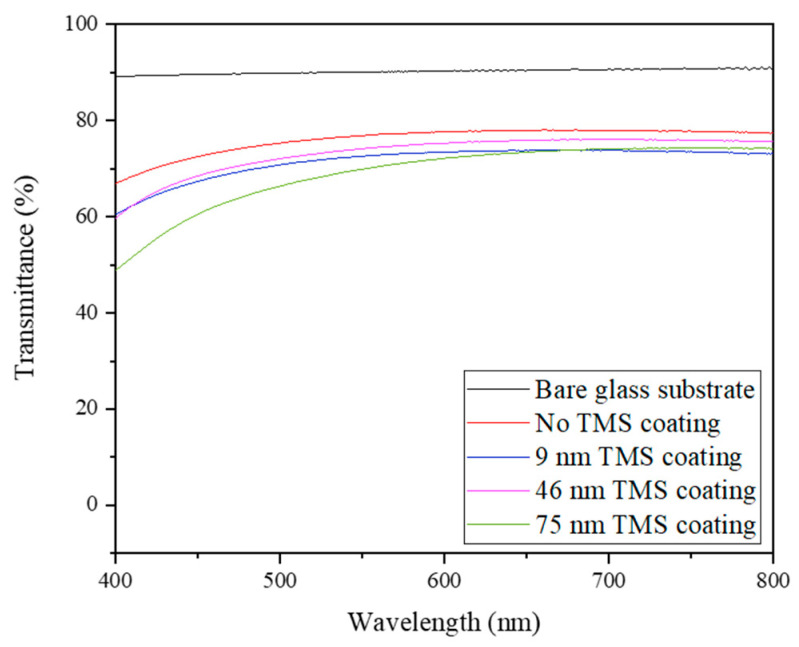
Optical transmittance of uncoated and TMS plasma-nanocoated AgNWs on glass with different coating thicknesses in comparison with the bare glass substrate.

For a glass-based electronic device, the optical transmittance of the device is one of the factors that influences the device’s overall performance. Therefore, the optical transmittance of the uncoated and TMS plasma-nanocoated AgNWs on glass substrates was measured in the wavelength range of 400 nm to 800 nm. In Figure 2, the optical transmittance of a bare glass is shown as the reference line, which was nearly 90%. With AgNWs sprayed onto the glass, the transmittance was reduced to ~67.1% at a 400 nm wavelength and ~77.4% at an 800 nm wavelength. After the TMS plasma nanocoating was deposited onto the AgNWs, the transmittance percentage decreased with the increase in plasma coating thickness. With 46 nm of TMS plasma nanocoating on, the optical transmittance changed to ~60.0% at a 400 nm wavelength, and ~75.7% at an 800 nm wavelength. Even with 75 nm of TMS plasma nanocoating on, the optical transmittance changed to 49.2% at 400 nm and remained ~74.2% at an 800 nm wavelength. The results shown in Figure 2 indicate that the TMS plasma nanocoating mainly affected the optical transmittance of AgNWs at lower wavelengths near 400 nm but showed negligible influence on the transparency of AgNWs at higher wavelengths above 600 nm.

### 3.2. Electrical Resistance Stability in Summer Season

**Figure 3:**
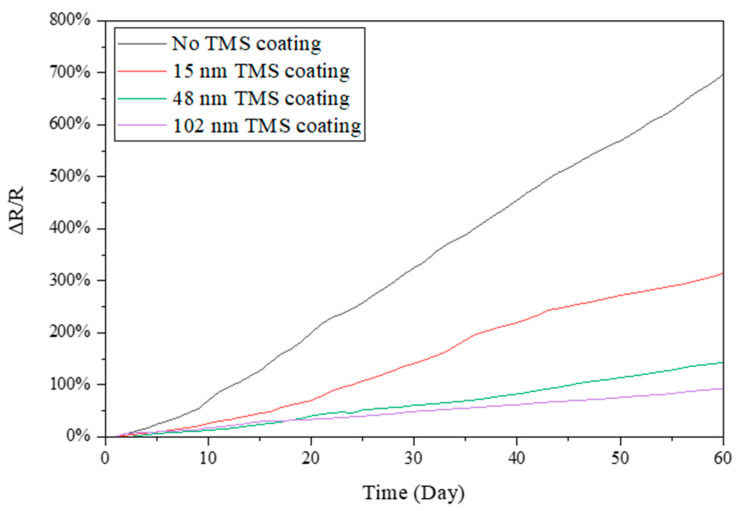
Electrical resistance changes in TMS plasma-nanocoated AgNWs on SEBS substrates stored in ambient air during summer with a lab room temperature of 20–22 °C and a relative humidity level of around 55–70% RH.

**Figure 4:**
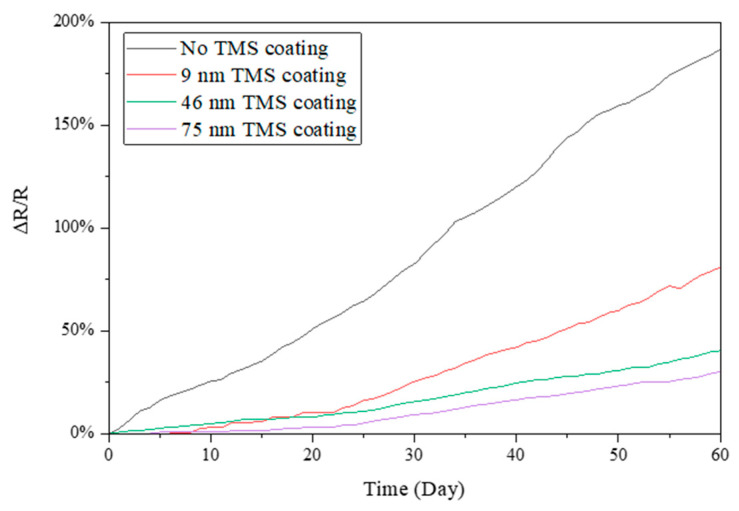
Electrical resistance changes in TMS plasma-nanocoated AgNWs on glass substrates stored in ambient air during summer with a lab room temperature of 20–22 °C and a relative humidity level of around 55–70% RH.

Electrical resistance was measured for the TMS plasma-nanocoated AgNWs that were stored in ambient air for 60 days during the summer season. In the summer, the lab room temperature was kept at 20–22 °C, with a relative humidity level of around 55–70% RH. As shown in Figure 3, without plasma coating, the resistance of the AgNWs increased rapidly with storage time. After 60 days of storage in ambient air, the resistance of the AgNWs on SEBS substrates increased by ~700% compared to the first-day measurement. In contrast, the TMS plasma-nanocoated AgNWs on SEBS showed a much slower increase in electrical resistance with time. It was observed that the higher the plasma coating thickness, the slower the increase in the electrical resistance. It was noted that the resistance increased by ~90% and ~140%, respectively, for the 48 nm and 102 nm TMS plasma-nanocoated AgNWs on SEBS. Figure 4 shows the resistance change in the TMS plasma-nanocoated AgNWs on glass. With no TMS coating, the electrical resistance of the AgNWs on glass increased by ~190% after 60 days of storage in ambient air as compared to the first-day measurement. With TMS plasma nanocoating on the AgNWs, the resistance increase was much slower than that of the uncoated AgNWs. With 46 nm and 75 nm TMS plasma nanocoatings, the resistance of the AgNWs on SEBS increased much less, by ~30% and ~40%, respectively, after 60 days of storage in ambient air.

### 3.3. Electrical Resistance Stability in Winter Season 

**Figure 5:**
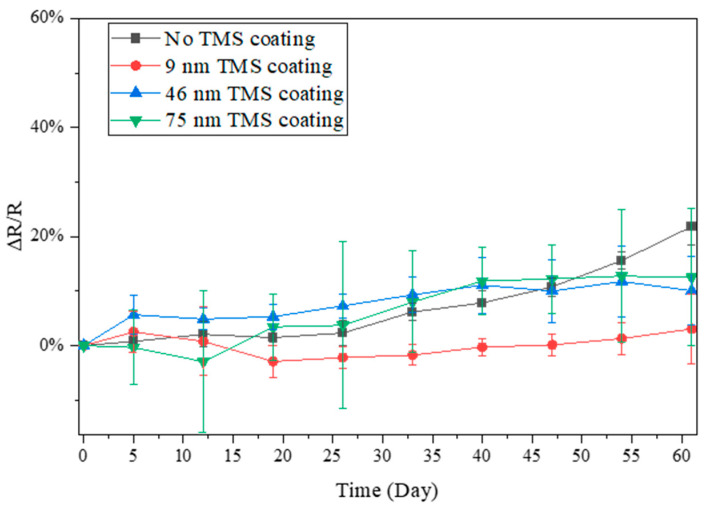
Electrical resistance changes in TMS plasma-nanocoated AgNWs on glass substrates stored in ambient air during winter with a lab room temperature of 20–22 °C and a relative humidity level of around 20–35% RH.

**Figure 6:**
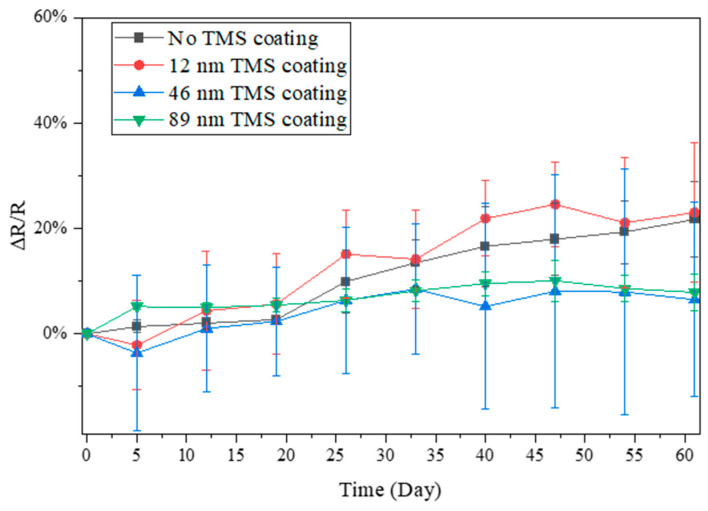
Electrical resistance changes in TMS plasma-nanocoated AgNWs on PLLA substrates stored in ambient air during winter with a lab room temperature of 20–22 °C and a relative humidity level of around 20–35% RH.

**Figure 7:**
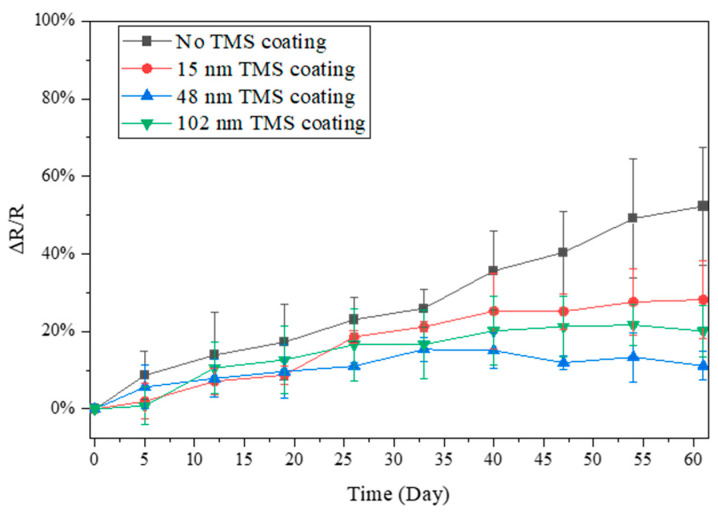
Electrical resistance changes in TMS plasma-nanocoated AgNWs on SEBS substrates stored in ambient air during winter with a lab room temperature of 20–22 °C and a relative humidity level of around 20–35% RH.

The resistance changes in the AgNWs stored in ambient air during the winter season were also monitored due to the much lower relative humidity (RH) in the air than in summer. During the winter, the lab room temperature was kept at 20–22 °C, with a relative humidity level of around 20–35% RH. From the results shown in Figure 5, Figure 6 and Figure 7, it was noted that the AgNWs on glass, SEBS, and PLLA showed a much slower increase with storage time in electrical resistance than in the summer season. After 60 days of storage in ambient air, the electrical resistance of the AgNWs on glass substrates increased by ~22%. In contrast, the TMS plasma-nanocoated AgNWs on glass substrates showed a slower increase of ~13%, with a 75 nm coating thickness (Figure 5). The electrical resistance of the AgNWs on PLLA substrates increased by ~22% and ~8%, respectively, for specimens without coating and with 89 nm TMS plasma nanocoatings (Figure 6). The electrical resistance of the AgNWs on SEBS substrates increased by about ~52% and 20%, respectively, for specimens without coating and with 102 nm TMS plasma nanocoatings (Figure 7). Due to the much lower resistance increase under low humidity levels and the overlapping large error bars in the figures, the passivation effects of the TMS plasma nanocoatings on the AgNWs were understated from the data shown in Figure 5, Figure 6 and Figure 7, but the results, overall, did show that the application of TMS plasma nanocoatings slowed down the electrical resistance increase in the AgNWs prepared on all the three substrates in the winter season with a low relative humidity in the ambient air.

### 3.4. Humidity Influence

**Figure 8:**
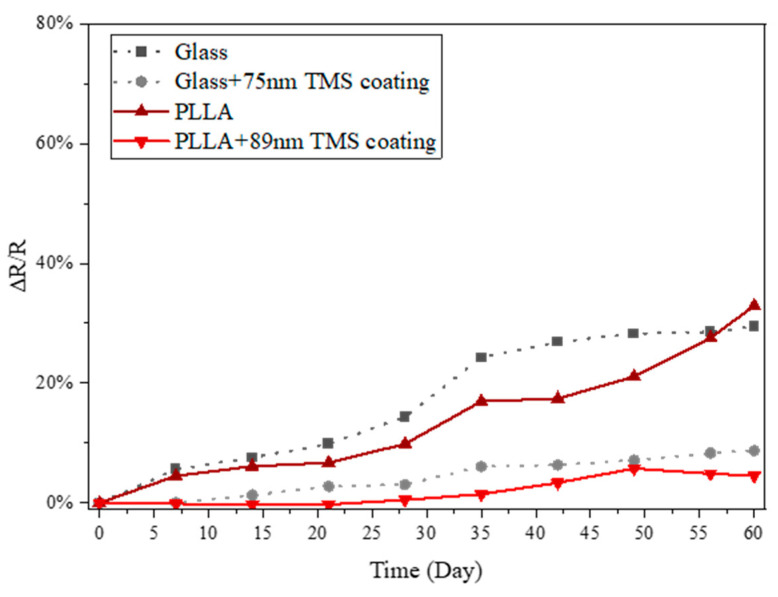
Electrical conductivity changes in uncoated and TMS plasma-nanocoated AgNWs on glass and PLLA substrates in a controlled environment at 21–22 °C and 40–55% relative humidity.

While the lab temperature was kept the same at 20–22 °C, the relative humidity in the ambient air varied significantly, with 55–70% RH in the summer and 20–35% RH in the winter. The substantially higher increase in the electrical resistance in the summer compared to the winter indicates that relative humidity is one of the critical factors that influence the surface oxidation of AgNWs. In this study, the AgNW samples were also stored in ambient air with the lab temperature kept at the same 20–22 °C, but with a relative humidity of 40–50% RH, which is an intermediate level of humidity between the winter season and summer season in the lab room. As shown in Figure 8, the electrical resistance increase in the AgNWs was found to be a little higher than that recorded in winter (Figure 5 and Figure 6) and much lower than that in summer (Figure 4). This result evidently indicates that relative humidity is a critical factor affecting the electrical stability of AgNWs. The data suggest a direct correlation between humidity levels and the degradation rate of AgNWs due to surface oxidation or corrosion, which in turn led to changes in electrical resistance. Higher humidity levels facilitate the oxidation/corrosion process, leading to a greater increase in resistance, whereas lower humidity levels slow down the oxidation/corrosion and thus result in a much lower increase in resistance.

**Figure 9:**
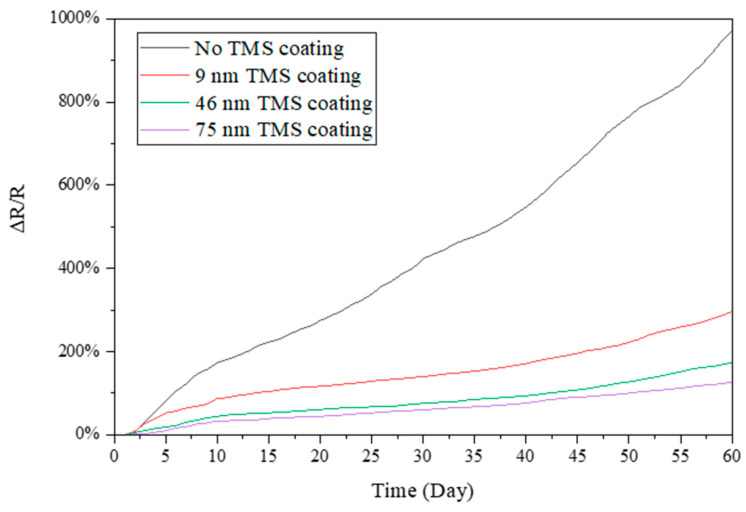
Electrical resistance changes in TMS plasma-nanocoated AgNWs on glass substrates stored in 37 °C PBS solution with different plasma coating thicknesses.

To further examine the effects of humidity, the electrical resistance change in the AgNWs on glass was also monitored by storing the test specimens in a 37 °C PBS solution as an accelerated condition to simulate the body temperature environment. As shown in Figure 9, without TMS plasma nanocoating, the AgNWs on glass stored in the PBS solution showed a sharply increased electrical resistance with storage time. After 60 days, the resistance of the non-TMS-coated group increased by ~970% compared to the initial resistance value. In contrast, with the TMS plasma coating applied, the increase rate of the resistance of the AgNWs was much suppressed. The TMS plasma-nanocoated AgNWs became much more stable with the thicker coating thicknesses of 46 nm and 75 nm. When 75 nm thick TMS plasma nanocoatings were applied to the AgNWs, the resistance increased only by ~130% after 60 days of storage in the 37 °C PBS solution.

### 3.5. Surface Morphology of TMS-Coated AgNWs on SEBS

**Figure 10:**
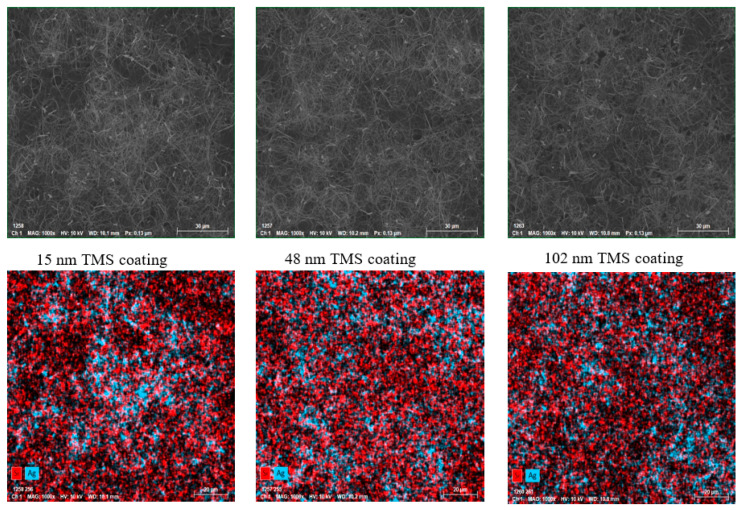
SEM images and EDS elemental mapping of Si element (the main chemical element in TMS plasma nanocoating, shown in red) and Ag element (the main chemical element in AgNWs, shown in blue) of TMS plasma-nanocoated AgNWs on SEBS substrates with different plasma coating thicknesses.

The surface morphology and element distribution of silicone and silver on the TMS plasma-nanocoated AgNWs on SEBS substrates were examined to determine if the TMS plasma nanocoatings fully covered the AgNWs. Figure 10 shows the SEM images and EDS elemental mapping of the Si element (the main chemical element in TMS plasma nanocoating, shown in red) and the Ag element (the main chemical element in AgNWs, shown in blue) of the TMS plasma-nanocoated AgNWs on SEBS substrates with different plasma coating thicknesses. On the EDS images in Figure 10, the red Si element occupies most parts of the images with uniform distribution, which indicates uniform TMS plasma nanocoatings deposited on the AgNWs. As the TMS plasma nanocoating thickness increased from 15 nm to 48 nm and 102 nm, the overall contents of the Si element (red) increased, while the Ag element contents (blue) were reduced.

### 3.6. Surface Wettability Change in TMS Plasma-Nanocoated AgNWs

**Figure 11:**
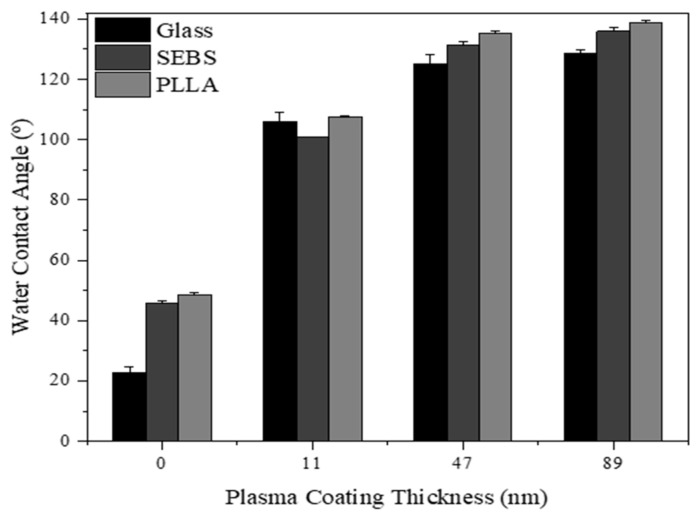
The coating thickness dependence of water surface contact angle of TMS plasma-nanocoated AgNWs on three different substrates.

Figure 11 shows the water surface contact angle change in the AgNWs before and after TMS plasma nanocoating application. For the AgNWs on glass, the water surface contact angles of the AgNWs on the three substrates were all less than 50 degrees, which indicates hydrophilic surfaces. With the TMS plasma nanocoatings applied with thicknesses from 11 nm to 89 nm, the surfaces of the AgNWs all had water surface contact angles higher than 100 degrees. In other words, the TMS plasma-nanocoated AgNWs on all the three substrates became hydrophobic. It was also noticed that the AgNWs’ surfaces became more hydrophobic with the increase in coating thickness of the TMS plasma nanocoatings.

## 4. Discussion

### 4.1. Effects of TMS Plasma Nanocoatings on AgNWs

The TMS plasma nanocoatings on AgNWs were found to have an influence on the electrical resistance and surface properties of the samples. The electrical resistance of the AgNWs on SEBS and glass substrates increased with thicker plasma coatings, i.e., a slight decrease in electrical conductivity (Figure 1). For the AgNWs on PLLA substrates, however, little changes in electrical resistance were observed after TMS plasma nanocoating deposition.

The application of TMS plasma nanocoatings slightly reduced the optical transparency of the AgNWs on glass substrates in the wavelength range of 400 nm to 800 nm (Figure 2). It was found that TMS plasma nanocoatings affected the optical transmittance of the AgNWs a little more at lower wavelengths near 400 nm but had a negligible influence on the transparency of the AgNWs at higher wavelengths above 600 nm. The optical transparency in the 400 to 800 nm range is important for applications in the visible light spectrum range, such as transparent electrodes in displays and solar cells. This study found that TMS plasma nanocoatings had a negligible effect on the optical transmittance of the AgNWs to ensure their desired optical transparency without compromising much on their electrical conductivity.

The application of TMS plasma nanocoatings also changed the surface wettability of the AgNWs. Without plasma coatings, the surfaces of the AgNWs were hydrophilic, with water surface contact angles of less than 50 degrees. In contrast, the TMS plasma-nanocoated AgNWs became hydrophobic. With thicker TMS plasma nanocoatings, the surfaces of the AgNWs were more hydrophobic (Figure 11). This change in surface wettability indicated that the TMS plasma nanocoatings had been successfully coated on the AgNWs surfaces.

The SEM images and EDS elemental mapping evidently show the successful application of TMS plasma nanocoatings onto the AgNWs’ surfaces (Figure 10). The EDS elemental mapping shows that there was a very little amount of exposed silver elements but a huge amount of silicone elements, which are the main elemental components of TMS plasma nanocoatings. This observation suggests that the AgNWs were uniformly covered by the TMS plasma nanocoatings, and the coverage became more complete with increasing coating thickness. These findings provide visual confirmation of the TMS plasma nanocoatings uniformly deposited on the AgNWs.

### 4.2. Surface Protection of AgNWs for Long-Term Stability

In this study, TMS plasma nanocoating was applied to AgNW surfaces to protect them from oxidation/corrosion and thus achieve long-term stability in terms of electronic conductivity. During the summer season with high humidity, the uncoated AgNWs on both SEBS and glass substrates showed rapid and dramatic increases in electrical resistance with storage time in ambient air (Figure 3 and Figure 4). In contrast, the TMS plasma-nanocoated AgNWs demonstrated significantly improved long-term stability with a much slower and lower increase in electrical resistance when stored in ambient air for up to 2 months. In other words, the uniform and dense TMS plasma nanocoatings deposited on the AgNW surfaces effectively protected them from oxidation by environmental moisture, thereby maintaining their long-term stability in electrical resistance or conductivity.

When stored in ambient air in the winter season with a much lower humidity level, the electrical resistance of the AgNWs on all three substrates, including glass, PLLA, and SEBS, increased at a much slower rate than in the summer season (Figure 5, Figure 6 and Figure 7). The results suggest that, with the room temperature controlled at 20–22 °C, humidity levels play a major role in the surface oxidation of AgNWs. With TMS plasma nanocoatings, the AgNWs on all the three substrates showed more stable conductivity compared to the uncoated AgNWs, although no significant differences were observed.

These findings indicated that the TMS plasma nanocoating provided a protective layer for the AgNWs, reducing their susceptibility to surface oxidation/corrosion, especially in a high-humidity environment and PBS solution. The TMS plasma nanocoating contributes to the long-term stability and functionality of AgNWs, making them suitable for various applications such as high-performance transparent electrodes or wearable electronic devices.

### 4.3. Factors Affecting the Stability of AgNWs

In this study, the influence of humidity on the electrical resistance changes in AgNWs was investigated by storing the uncoated and TMS plasma-nanocoated samples at room temperature but at different humidity levels. Previous studies have highlighted the impact of natural light illumination, humidity levels, and temperature on the electrical resistance of AgNW films [28]. It has been observed that storing AgNW films in dry air can prolong the devices’ lifetime, while the application of water-proofing layers on AgNW films can enhance their stability [17,29,30,31,32]. However, some studies have reported that high relative humidity (90% RH) does not play a critical role in the corrosion process of AgNWs [17,33,34,35]. In this study, significant differences were observed in the resistance increase in AgNWs between the summer and winter seasons, indicating that humidity did significantly affect the surface oxidation of AgNWs.

It is important to note that, during the summer season, both elevated temperatures and relative humidity coexist. In this study, the electrical resistance change in the AgNWs on glass was also examined by storing the test specimens in a 37 °C PBS solution as an accelerated condition to simulate the body temperature environment (Figure 9). It was also found that the TMS plasma nanocoatings also effectively protected the AgNWs from degradation and maintained a very stable electrical resistance or conductivity.

### 4.4. Protection Mechanism of AgNWs by TMS Plasma Nanocoatings

AgNWs are prone to oxidation and sulfidation when exposed to air, resulting in a deterioration of electrical properties. The metal corrodes due to chemical reactions between its surface and the hydrogen sulfide, water, or carbonyl sulfide (OCS) in the air [36,37,38,39,40,41]. The formation of sulfide on AgNWs is triggered by the presence of water and nitrogen dioxide, which highlights the unavoidable corrosion reaction of silver. To address this issue, it is crucial to develop a thin protective coating that shields the AgNWs’ surfaces from corrosion while preserving their electrical and optical properties.

In this study, TMS plasma nanocoatings with varying thicknesses were uniformly applied to the surfaces of the AgNWs. The TMS plasma nanocoatings are pinhole free, highly cross-linked, chemically stable, and excellent barriers, and have a strong adhesion to various substrates, including metallic materials [42]. First, the TMS plasma coatings served as a barrier that effectively shielded the AgNWs from moisture in the ambient air and the aqueous PBS solutions, and, consequently, significantly reduced the oxidation/corrosion rate and improved the stability of the AgNWs.

**Figure 12:**
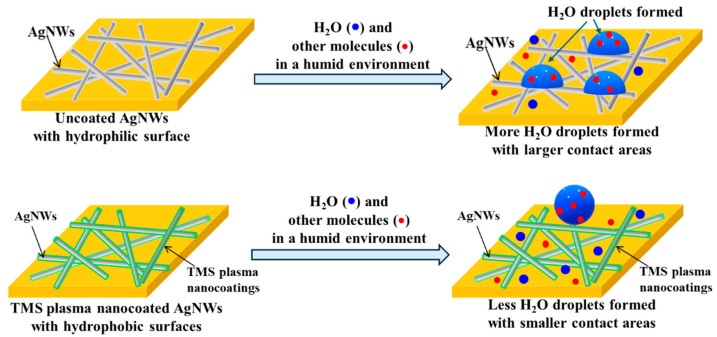
A schematic of plausible protection mechanism diagram of AgNWs by TMS plasma nanocoatings.

The TMS plasma nanocoatings altered the surface wettability of the AgNWs, transforming them from hydrophilic to very hydrophobic or nearly superhydrophobic. By creating a very hydrophobic surface, the moisture in the surrounding atmosphere was hindered from forming water droplets on the AgNWs’ surfaces. As schematically shown in Figure 12, thereby, the hydrophobic surfaces of TMS plasma-nanocoated AgNWs limit the adhesion of water droplets to and direct water contact with AgNWs and thus prevent them from rapid oxidation/corrosion and the deterioration of electrical properties. The results obtained in this study indicate that the TMS plasma nanocoatings could serve as an alternative method to enhance the long-term stability of AgNWs and help maintain the AgNW-based device’s overall performance and functionality. The novelty of the present work is that the TMS plasma nanocoatings not only provided suitable passivation of the AgNWs, but also made their surfaces very hydrophobic to further enhance their chemical stability and electrical stability in a humid environment.

## 5. Conclusions

This study experimentally investigated TMS plasma nanocoatings to passivate AgNWs and thus improve their electrical stability. The results showed that the application of TMS plasma nanocoatings onto AgNWs had a negligible effect on their electrical conductivity and optical transparency, especially at higher wavelengths above 600 nm. Under different humidity levels, the TMS plasma nanocoatings significantly improved the long-term electrical stability of AgNWs prepared on various substrates, including glass, porous SEBS, and PLLA. The results evidently demonstrate that TMS plasma nanocoatings are effective in protecting AgNWs from degradation through oxidation/corrosion in high-humidity environments in summer or even with prolonged immersion in an aqueous PBS solution for up to 2 months. This study also revealed that humidity is the primary factor influencing the degradation of AgNWs and, consequently, their increase in resistance. These findings highlight the potential of TMS plasma nanocoatings in extending the lifespan and functionality of AgNWs as transparent electrodes for on-skin devices. Required future studies will include detailed characterizations using thermogravimetric analysis (TGA), X-ray photoelectron spectroscopy (XPS), and X-ray diffraction (XRD) to provide a more comprehensive understanding of the nanocoating’s impact on AgNWs. Post-stability characterization using electrochemical impedance spectroscopy (EIS), XRD, and EDAX analysis will be essential to further prove the stabilization of AgNWs by TMS plasma nanocoatings. It will also be important to explore the potential correlation between surface wettability measurements and durability indices for TMS plasma-nanocoated AgNWs.

## Data Availability

Not applicable.
